# Unlocking the roles of plasma soluble T-cell immunoglobulin and mucin domain-containing protein 3 in kidney diseases: findings from native and allograft biopsy cohorts

**DOI:** 10.1186/s43556-026-00469-6

**Published:** 2026-05-27

**Authors:** Yamei Li, Hua Zhang, Yangjuan Bai, Huan Xu, Yan Luo, Dan Ye, Xueqiao Wang, Xingxin Gong, Qu Yang, Hanjing Liu, Binqi Yang, Zheyuan Zhang, Yuxin Ye, Yunfei An, Xinhua Dai, Lanlan Wang, Yunying Shi

**Affiliations:** 1 Department of Laboratory Medicine/Clinical Laboratory Medicine Research Center, West China Hospital, Sichuan University, No.37 Guoxue Xiang, Wuhou District, Chengdu, Sichuan Province 610041 China; 2 Sichuan Clinical Research Center for Laboratory Medicine, Chengdu, China; 3 Department of Pathology, General Hospital of Western Theater Command, Chengdu, Sichuan Province China; 4 Tissue Stress Injury and Functional Repair Key Laboratory of Sichuan Province, General Hospital of Western Theater Command, Chengdu, Sichuan Province China; 5 College of Medicine, Southwest Jiaotong University, Chengdu, China; 6 Department of Nephrology, West China Hospital, Sichuan University, No.37 Guoxue Xiang, Wuhou District, Chengdu, Sichuan Province 610041 China

**Keywords:** Soluble TIM-3, Biomarker, Kidney transplantation, Chronic kidney diseases, Tubulointerstitial fibrosis, Tubular atrophy

## Abstract

Chronic kidney disease (CKD) and renal allograft failure represent escalating global health burdens characterized by progressive tissue remodeling. Although interstitial fibrosis and tubular atrophy (IF/TA) are the primary determinants of long-term prognosis, their assessment currently relies on invasive kidney biopsies, which are unsuitable for longitudinal monitoring. Here, we identify soluble T-cell immunoglobulin and mucin domain-containing protein 3 (sTIM-3) as a robust, non-invasive indicator of renal histopathology and clinical outcomes. In this multi-cohort study, we enrolled 256 kidney transplant recipients (KTRs) with allograft biopsies, 442 native CKD patients with biopsy-confirmed diagnoses, and 44 KTRs with longitudinal follow-up. Pre-biopsy plasma sTIM-3 levels were quantified by ELISA and analyzed for associations with renal function, histopathological parameters, and adverse outcomes. We found that elevated sTIM-3 levels consistently correlated with impaired renal function across diverse etiologies. Notably, post-transplantation sTIM-3 exhibited slower decline kinetics compared to estimated glomerular filtration rate (eGFR), suggesting that it may reflect active tissue remodeling rather than transient functional shifts. Histopathological analyses revealed that sTIM-3 levels increased progressively with IF/TA severity, demonstrating good discriminative capacity for moderate-to-severe fibrosis. Furthermore, in KTRs, elevated sTIM-3 predicted both allograft failure and rapid eGFR decline, with its prognostic value for the latter persisting after adjustment for eGFR and urine protein. Collectively, these findings establish sTIM-3 as a non-invasive biomarker reflecting tubulointerstitial fibrosis and adverse outcomes across diverse kidney disease settings, offering complementary information to conventional functional markers.

**Supplementary Information:**

The online version contains supplementary material available at 10.1186/s43556-026-00469-6.

## Introduction

Chronic kidney disease (CKD), including both native kidney disorders and post-transplant complications, represents an escalating global health challenge that frequently advances to end-stage renal disease. The central pathological hallmark driving this progression is interstitial fibrosis and tubular atrophy (IF/TA), which is considered the common final pathway of diverse renal injuries, leading to irreversible functional decline [1]. While kidney biopsy remains the gold standard for assessing the severity of IF/TA and other pathological lesions, its invasiveness, sampling limitations, and unsuitability for repeated monitoring hinder its widespread clinical utility. This underscores the critical need for reliable, non-invasive biomarkers that can accurately reflect underlying fibrotic activity and longitudinal pathological changes.

In current clinical practice, kidney status is primarily monitored through glomerular function and damage-related markers, such as serum creatinine (SCR), estimated glomerular filtration rate (eGFR) and urine protein. However, these conventional indicators often demonstrate a “blind spot” during early disease stages, failing to capture significant histopathological damage until substantial nephron loss has occurred. Moreover, eGFR and proteinuria provide limited specificity regarding actual tissue-level remodeling and interstitial scarring that dictate long-term prognosis. Consequently, identifying novel non-invasive biomarkers that bridge the gap between functional assessment and structural histopathology is paramount for refining risk stratification and for guiding therapy targeting kidney fibrosis.

T-cell immunoglobulin and mucin domain-containing protein 3 (TIM-3), a type I transmembrane protein originally identified as an immune checkpoint regulator, has emerged as a key mediator in inflammatory and fibrotic environments [2–5]. Within the renal context, TIM-3 is constitutively expressed on proximal tubule epithelial cells and immune infiltrates, where it regulates podocyte and tubulointerstitial injury in various animal models [6–8]. Its soluble form, sTIM-3, is generated via proteolytic ectodomain shedding by a disintegrin and metalloproteinases 10 and 17 (ADAM10/17) [9], and has shown diagnostic potential in malignancies and infectious diseases [10–12]. Our previous preliminary investigations demonstrated that elevated sTIM-3 positively correlates with allograft dysfunction in kidney transplant recipients (KTRs) [13, 14]. However, several critical knowledge gaps remain unexplored, particularly whether this association is unique to the alloimmune milieu or represents a broader pathological response in native CKD, and whether sTIM-3 can serve as a robust surrogate for specific histological lesions such as IF/TA.

Therefore, this study aimed to systematically evaluate the clinical potential of plasma sTIM-3 as a comprehensive renal biomarker across a spectrum of kidney diseases. We hypothesized that sTIM-3 levels reflect the severity of renal parenchymal damage and possess prognostic value independent of traditional markers. To this end, we conducted a multi-cohort study comprising a biopsy-proven KTR cohort, a biopsy-proven native CKD cohort, and a longitudinal KTR cohort (KTR-dyna) for dynamic monitoring. Plasma sTIM-3 concentrations were quantified to assess their associations with clinical laboratory markers, detailed histopathological parameters (e.g., IF/TA, arteriolar sclerosis, and inflammation), and adverse outcomes. Our findings demonstrate that elevated sTIM-3 is significantly associated with impaired renal function, independently correlates with IF/TA severity, and serves as a potent predictor of graft failure and rapid eGFR decline in KTRs. These results collectively highlight the diagnostic and prognostic significance of sTIM-3 in both native and transplant-related kidney diseases.

## Results

### Patient characteristics

**Fig. 1:**
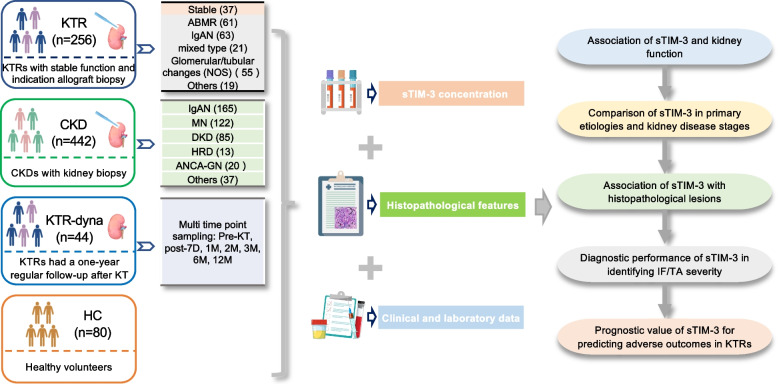
Flowchart of study population and study protocol

A total of 822 participants were enrolled across four cohorts, including 256 KTRs with stable graft function or undergoing indication allograft biopsy, 442 native CKD patients undergoing kidney biopsy, 44 KTRs with one-year regular follow‑up after transplantation (KTR‑dyna), and 80 healthy controls (HCs). Plasma sTIM‑3 concentrations, clinical and laboratory data, and histopathological features were systematically collected to evaluate the association of sTIM‑3 with kidney function, its diagnostic performance for IF/TA severity, and its prognostic value for adverse outcomes in KTRs. The overall study design and cohort allocation are summarized in Fig. 1.

Demographic and clinical characteristics of the KTR, CKD and KTR-dyna cohorts, as well as HCs, are summarized in Table 1. In the KTR and KTR-dyna cohorts, the median ages were 37 and 33 years, with 73.04% and 75.00% being male, respectively. Living donors constituted the majority in both groups (75.00% and 77.27%). In contrast, the CKD (median age: 47 years) and HC (median age: 42 years) cohorts were older and had a balanced gender distribution. Among KTRs, the median post-transplant time was 3.15 years, the median number of human leukocyte antigen (HLA) mismatches was 4 (IQR 3—6), and the median trough tacrolimus concentration was 5.42 ng/mL. Overall, the KTR cohort exhibited worse kidney function and a higher neutrophil-to-lymphocyte ratio than the CKD and HC groups. Detailed demographic and clinical characteristics for the antibody-mediated rejection (ABMR), new onset or recurrence of IgA nephropathy (IgAN), membranous nephropathy (MN), native IgAN and diabetic kidney disease (DKD) subgroups are provided in Table S1.

**Table 1:** Baseline characteristics of study population

Characteristic	KTR cohort	CKD cohort	KTR-dyna cohort	HC
Number	256	442	44	80
Age (year)	37 (31—47)	47 (33—56)	33 (27—37)	42 (39—49)
Male, n (%)	187 (73.04%)	241 (54.52%)	33 (75.00%)	37 (46.25%)
Living donor, n (%)	192 (75.00%)	NA	34 (77.27%)	NA
HLA-mismatches (A, B, DR, DQ)	4 (3—6)	NA	4 (3—5)	NA
Post-transplant time (years)	3.15 (1.20—6.19)	NA	NA	NA
Trough concentration of tacrolimus (ng/mL)	5.42 (4.65—6.54)	NA	NA	NA
SCR (μmol/L)	156.00 (110.50—214.00)	100.00 (72.00—159.00)	NA	70.00 (59.50—81.00)
eGFR (mL/min/1.73m2)	44.66 (30.19—66.28)	70.49 (39.46—98.35)	NA	103.36 (91.93—110.61)
Serum urea (mmol/L)	9.60 (6.80—13.50)	6.40 (4.90—9.90)	NA	4.70 (4.10—5.40)
Serum cystatin C (mg/L)	1.86 (1.38—2.52)	1.19 (0.97—1.73)	NA	0.81 (0.73—0.85)
Urine protein (n)				
0	63	15	NA	80
±	29	22	NA	0
+	47	72	NA	0
+ +	76	159	NA	0
+ + +	32	102	NA	0
+ + + +	9	72	NA	0
UPCR*	0.16 (0.07—0.38)	0.18 (0.09—0.42)	NA	ND
ALT (IU/L)	14 (9—19)	16 (11—24)	NA	19 (14—27)
AST (IU/L)	16 (12—19)	18 (15—23)	NA	20 (17—23)
Total cholesterol (mmol/L)	5.11 (4.35—5.96)	4.83 (4.12—6.01)	NA	4.47 (4.18—4.88)
Triglyceride (mmol/L)	1.71 (1.21—2.31)	1.72 (1.28—2.61)	NA	1.01 (0.77—1.29)
Hemoglobin (g/L)	126.72 ± 25.35	125.21 ± 22.82	NA	143.36 ± 14.74
Neutrophil-to-lymphocyte ratio	3.18 (2.15—5.41)	2.49 (1.83—3.45)	NA	1.78 (1.48—2.05)

*HLA* human leukocyte antigen, *SCR* serum creatinine, *eGFR* estimated glomerular filtration rate, *UPCR* Urine protein-to-creatinine ratio, *ALT* Alanine aminotransferase, *AST* Aspartate aminotransferase, *KTR* kidney transplant recipient, *CKD* chronic kidney disease, *NA* not applicable, *ND* not detected. * In the KTR cohort, UPCR was missing in 66.8% of patients; In the CKD cohort, UPCR was missing in 2.3% of patients

### Association of plasma sTIM-3 with renal function

**Fig. 2:**
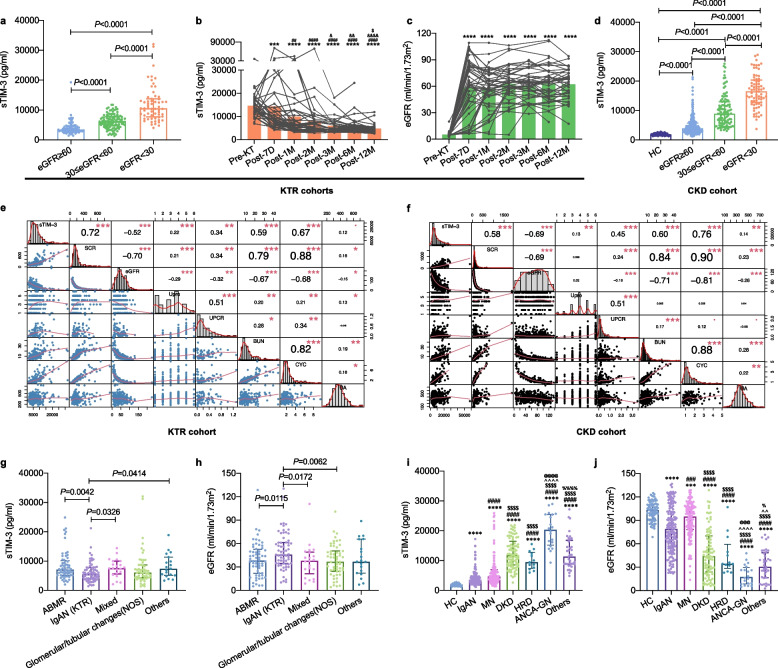
Association between sTIM-3 levels and renal function, and different renal pathological types in the KTR and native CKD cohorts. **a**, **d** sTIM-3 concentrations across different renal function subgroups in KTRs (**a**) and in native CKD patients (**d**); **b**—**c** Longitudinal monitoring of sTIM-3 concentrations (**b**) and eGFR levels (**c**) at pre-transplant and post-transplant time points; **e**—**f**. Correlation matrices of sTIM-3 levels with renal function biomarkers in KTRs (**e**) and in native CKD patients (**f**); **g**, **i** sTIM-3 levels in allograft dysfunction subgroups (**g**) and CKD subgroups (**i**); **h**, **j** Corresponding eGFR levels in allograft dysfunction subgroups (**h**) and CKD subgroups (**j**). Abbreviations: SCR, serum creatinine; Upro, urine protein; UPCR, urine protein-to-creatinine ratio; BUN, blood urea nitrogen; CYC, Cystatin C; UA, uric acid; ABMR, antibody-mediated rejection; IgAN (KTR), new onset or recurrence of IgA nephropathy; mixed, mixed pathological types; NOS, not otherwise specified; HC, health control; MN, membranous nephropathy; DKD, diabetic kidney disease; HRD, hypertensive renal disease; ANCA-GN, anti-neutrophil cytoplasmic antibody-associated glomerulonephritis. Note: In Fig. 2**b** and **c**, ***P < 0.001, ****P < 0.0001 vs. Pre-KT; ^##^*P* < 0.01, ^####^*P* < 0.0001 vs. post-7D; ^&^*P* < 0.05, ^&&^*P* < 0.01, ^&&&&^*P* < 0.0001 vs. post-1 M; ^$^*P* < 0.05 vs. post-2 M. In Fig. 2**i** and **j**, ***P < 0.001, ****P < 0.0001 vs. HC; ^###^*P* < 0.001, ^####^*P* < 0.0001 vs. IgAN; ^$$$$^*P* < 0.0001 vs. MN; ^^^^*P* < 0.01, ^^^^^^*P* < 0.0001 vs. DKD; ^@@@^*P* < 0.001, ^@@@@^*P* < 0.0001 vs. HRD; ^%^*P* < 0.05, ^%%%%^*P* < 0.0001 vs. ANCA-GN

To validate the generalizability of our previous findings [13, 14], we examined the correlations between sTIM-3 levels and renal function markers in both transplant and non-transplant settings. Patients were divided into three subgroups based on eGFR thresholds of 60 and 30 mL/min/1.73m^2^. We observed that sTIM-3 levels increased progressively with the deterioration of kidney function in both KTR and native CKD cohorts (Fig. 2a and d). Correlation analyses confirmed that sTIM-3 levels were significantly correlated with renal function markers, with coefficients ranging from 0.34 to 0.72 in the KTR cohort and 0.45 to 0.76 in the CKD cohort (Fig. 2e and f). Notably, this correlation remained consistent when stratified by primary etiologies, including ABMR (r = 0.54—0.77), new onset or recurrence of IgAN (r = 0.48—0.72), native IgAN (r = 0.32—0.68), MN (r = 0.39—0.78), and DKD (r = 0.26—0.79) (Fig. S1). Additionally, in the non-overlapping longitudinal KTR-dyna cohort (*n* = 44), sTIM-3 concentration was 13,575.96 (11,434.72—17,005.82) pg/mL pre-transplantation, and decreased significantly to 8356.95 (6444.39—12,186.53) pg/mL by day 7 post-transplantation. These concentrations continued to decline and stabilized by 6 months [4766.27 (3637.99—5591.69) pg/mL] (Fig. 2b). In contrast, eGFR increased significantly at day 7 and remained stable thereafter (Fig. 2c). These data suggest that sTIM-3 may serve as a universal biomarker of renal function, applicable to both native CKD and KTR populations.

### Plasma sTIM-3 levels across renal pathologies and disease stages

To explore the pathophysiological relevance of sTIM-3, we compared its levels across various etiologies of allograft dysfunction and native CKD. In the KTR cohort, patients with new onset or recurrence of IgAN showed significantly lower sTIM-3 levels but higher eGFR than those with ABMR or mixed etiologies (Fig. 2g-h). In the native CKD cohort, plasma sTIM-3 was significantly elevated across all pathological types compared to HCs. Among the various etiologies, patients with anti-neutrophil cytoplasmic antibody-associated glomerulonephritis (ANCA-GN), hypertensive renal disease (HRD) and DKD exhibited the most pronounced increases in sTIM-3, which inversely mirrored the eGFR patterns observed across these groups (Fig. 2i-j). Notably, MN patients showed significantly higher sTIM-3 and eGFR than IgAN patients (Fig. 2i-j). Further analysis revealed that sTIM-3 and eGFR levels remained comparable across different stages of MN (Fig. S2b and S2e). In contrast, in native IgAN and DKD, sTIM-3 increased in a stepwise manner that correlated with declining eGFR (Fig. S2a, S2c, S2d and S2f). Collectively, these data from multiple independent subcohorts validate the robust inverse association between plasma sTIM-3 and eGFR, reinforcing its potential as a reliable biomarker reflecting renal function across diverse kidney disease settings.

### Association of plasma sTIM-3 with kidney histological features

**Fig. 3:**
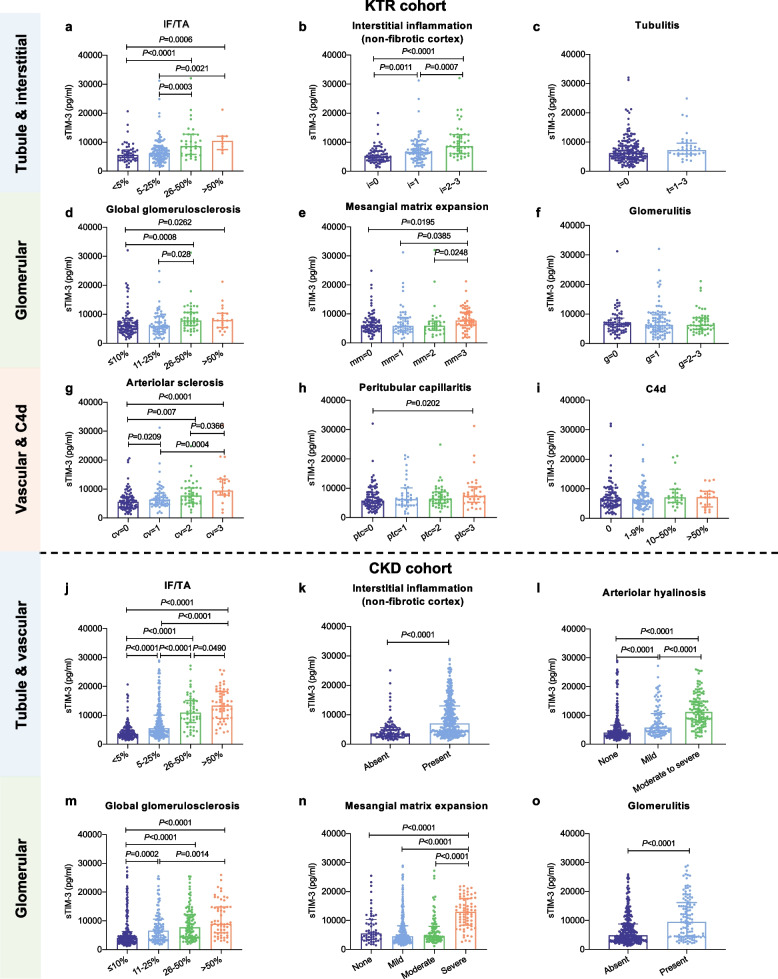
Association between plasma sTIM-3 and kidney histological lesion features. The scatter plots depict variations in sTIM-3 levels across different severities of allograft histological lesions in KTRs, including IF/TA (**a**), interstitial inflammation (non-fibrotic cortex) (**b**), tubulitis (**c**), global glomerulosclerosis (**d**), mesangial matrix expansion (**e**), glomerulitis (**f**), arteriolar sclerosis (**g**), peritubular capillaritis (**h**) and C4d deposition (**i**). Association of plasma sTIM-3 and kidney histological lesions in native CKD patients, including IF/TA (**j**), interstitial inflammation (non-fibrotic cortex) (**k**), arteriolar hyalinosis (**l**), global glomerulosclerosis (**m**), mesangial matrix expansion (**n**) and glomerulitis (**o**)

To elucidate the link between sTIM-3 and kidney histological lesion features, we systematically investigated the association between sTIM-3 levels and various histopathological parameters in both the KTR and native CKD cohorts. In KTRs, sTIM-3 levels increased progressively with advancing severity of IF/TA, interstitial inflammation (non-fibrotic cortex), global glomerulosclerosis and arteriolar sclerosis. However, no significant associations were observed with tubulitis, glomerulitis, peritubular capillaritis or C4d deposition (Fig. 3a-i). Similarly, in the native CKD cohort, sTIM-3 levels were significantly stratified by the severity of histopathological changes across all renal compartments. Specifically, sTIM-3 concentrations increased progressively in parallel with advancing grades of IF/TA, interstitial inflammation (non-fibrotic cortex), arteriolar hyalinosis, global glomerulosclerosis, mesangial matrix expansion and glomerulitis (Fig. 3j-o). Subgroup analyses further confirmed that sTIM-3 consistently correlated with IF/TA and glomerulitis across IgAN, MN, and DKD etiologies (Fig. S3). These data identify sTIM-3 as a multifaceted biomarker that mirrors the burden of chronic kidney lesions, most notably IF/TA, regardless of the underlying pathology.

### Evaluation of sTIM-3 performance in identifying kidney fibrosis

IF/TA represents the primary histological determinant of chronic kidney damage. Given the significant correlations among sTIM-3, eGFR and IF/TA severity (Fig. 2e-2f and Fig. S4a-S4n), we employed univariable and multivariable generalized linear regression models to determine whether sTIM-3 independently associates with IF/TA in KTR and CKD patients. Multivariable analyses showed that sTIM-3 levels (B = 0.002, *P* < 0.0001) and the sTIM-3 × eGFR interaction (B = −6.205 × 10^–5^, *P* = 0.0022) were independently associated with IF/TA areas in KTRs (Table 2). Similarly, in the CKD cohort, sTIM-3 levels (B = 0.0015, *P* < 0.0001) and the sTIM-3 × eGFR interaction (B = −2.792 × 10^–5^, *P* < 0.0001) remained independent correlates with IF/TA area, alongside total protein, hemoglobin and creatine kinase (Table 2). These findings demonstrate that sTIM-3 is a robust indicator of IF/TA across both transplant and native kidney disease settings. Notably, this relationship is significantly modulated by renal function, as evidenced by the consistent sTim‑3 × eGFR interaction.

**Table 2:** Univariable and multivariable generalized linear regression analyses evaluating the association between sTIM-3 and IF/TA in KTR and CKD cohorts

Variables	KTR cohort				CKD cohort			
	**Univariable regression**		**Multivariable regression**		**Univariable regression**		**Multivariable regression**	
	**B (95%CI)**	***P***	**B (95%CI)**	***P***	**B (95%CI)**	***P***	**B (95%CI)**	***P***
Age (years)	−0.012 (−0.184, 0.160)	0.889			0.042 (−0.102, 0.185)	0.568		
Female	−2.021 (−6.664, 2.622)	0.394			−5.669 (−9.627, −1.711)	**0.005**	−4.971 (−8.889, −1.054)	**0.013**
Post-transplant time (years)	0.574 (0.085, 5.300)	**0.021**	0.647 (−0.407,1.702)	0.229	NA	NA	NA	NA
Deceased donor	0.759 (−3.922, 0.101)	0.751			NA	NA	NA	NA
sTIM-3 (pg/mL)	0.001 (0.0006, 0.0015)	** < 0.0001**	0.002 (0.001, 0.004)	** < 0.0001**	0.001 (0.0001, 0.0011)	** < 0.0001**	0.0015 (0.0011,0.0019)	** < 0.0001**
sTIM-3 × eGFR interaction effect	−4.179 × 10^–5^ (−8.865 × 10^–5^, 5.078 × 10^–6^)	**0.081**	−6.205 × 10^–5^ (−0.0001, −2.232 × 10^–5^)	**0.0022**	−1.334 × 10^–5^ (−2.167 × 10^–5^, −5.010 × 10^–6^)	**0.0017**	−2.792 × 10^–5^ (−3.606 × 10^–5^, −1.979 × 10^–5^)	** < 0.0001**
Uric acid (μmol/L)	0.002 (−0.019, 0.022)	0.888			0.046 (0.026, 0.066)	** < 0.0001**	0.017 (−0.002, 0.036)	0.085
Total protein (g/L)	−0.555 (−0.823, −0.287)	** < 0.0001**	−0.384 (−0.972, 0.205)	0.201	0.228 (0.045, 0.411)	**0.015**	0.307 (0.140, 0.473)	**0.0003**
Triglyceride (mmol/L)	0.655 (−1.204, 2.514)	0.490			−0.221 (−1.492, 1.051)	0.734		
Creatine kinase (U/L)	−0.029 (−0.092, 0.034)	0.371			0.029 (0.012, 0.046)	**0.001**	0.023 (0.008, 0.037)	**0.002**
Lactate dehydrogenase (U/L)	0.032 (−0.003, 0.067)	**0.073**	−0.024 (−0.094, 0.046)	0.507	0.012 (−0.024, 0.048)	0.524		
Hemoglobin (g/L)	−0.154 (−0.230, −0.079)	** < 0.0001**	0.147 (−0.046, 0.339)	0.136	−0.282 (−0.365, −0.199)	** < 0.0001**	−0.121 (−0.217, −0.025)	**0.014**
Platelet count (×10^9^/L)	0.014 (−0.015, 0.042)	0.340			0.002 (−0.023, 0.027)	0.874		
White blood cell (×10^9^/L)	0.113 (−0.520, 0.746)	0.726			0.261 (−0.529, 1.051)	0.517		
Neutrophil-to-lymphocyte ratio	0.177 (−0.116, 0.470)	0.236			0.855 (0.067, 1.644)	**0.033**	0.080 (−0.626, 0.787)	0.823
Trough concentration of tacrolimus (ng/mL)	−0.365 (−1.392, 0.663)	0.487			NA	NA	NA	NA

*KTR* kidney transplant recipients, *CKD* chronic kidney disease, *eGFR* estimated glomerular filtration rate, *95% CI* 95% confidence interval, *NA* not applicable

To evaluate the diagnostic performance of sTIM‑3 across different stages of renal fibrosis, we conducted receiver-operating characteristic (ROC) curve analysis in both the KTR and CKD cohorts. IF/TA was categorized into four stages (0—3) based on the fibrotic area (< 5%, 5%—25%, 26%—50%, and > 50%, respectively). In the KTR cohort, sTIM-3 demonstrated significant discriminative capacity, with area under the curves (AUCs) increasing alongside fibrosis severity: 0.61 (95%CI: 0.53—0.70, *P* = 0.014) for stage 0 vs. 1—3; 0.74 (95%CI: 0.66—0.82, *P* < 0.0001) for stages 0—1 vs. 2—3; and 0.79 (95%CI: 0.67—0.90, *P* = 0.006) for stages 0—2 vs. 3. The optimal cutoffs, determined by the Youden index, were 4,960.83, 7,857.85, and 6,161.10 pg/mL, respectively. When sensitivity was fixed at approximately 90%, sTIM-3 cutoffs exhibited high clinical utility for ruling out advanced fibrosis, although specificity remained low. Conversely, setting specificity at 90% provided higher thresholds (9,492.20 to 12,559.83 pg/mL) suitable for confirming severe IF/TA (Table 3).

**Table 3:** Diagnostics performance of sTIM-3 for assessing kidney fibrosis in KTRs and CKD patients with kidney biopsies

	**Fibrosis stage** **Non-event vs event**	**Prevalence of event**	**AUC (95%CI)**	***P* value**	**Cutoff criteria**	**Cutoff (pg/mL)**	**Sensitivity**	**Specificity**	**PLR**	**NLR**
KTRs	0 vs 1—3	75.8%	0.61 (0.53—0.70)	0.014	Youden’s index	4960.83	0.75	0.48	1.45	0.52
					Sensitivity = 90%	3153.55	0.90	0.08	0.98	1.29
					Specificity = 90%	9492.20	0.26	0.90	2.66	0.82
	0—1 vs 2—3	19.1%	0.74 (0.66—0.82)	< 0.0001	Youden’s index	7857.85	0.68	0.76	2.87	0.42
					Sensitivity = 90%	5137.84	0.90	0.38	1.46	0.26
					Specificity = 90%	10,603.75	0.34	0.90	3.44	0.73
	0—2 vs 3	3.7%	0.79 (0.67—0.90)	0.006	Youden’s index	6161.10	1.00	0.498	1.99	0.00
					Sensitivity = 90%	6194.43	0.88	0.50	1.74	0.25
					Specificity = 90%	12,559.83	0.13	0.90	1.28	0.97
CKDs	0 vs 1—3	70.1%	0.77 (0.73—0.82)	< 0.0001	Youden’s index	6766.99	0.56	0.88	4.78	0.50
					Sensitivity = 90%	2963.87	0.90	0.37	1.43	0.27
					Specificity = 90%	7573.63	0.11	0.90	4.98	0.55
	0—1 vs 2—3	23.6%	0.82 (0.77—0.86)	< 0.0001	Youden’s index	8243.67	0.78	0.78	3.52	0.28
					Sensitivity = 90%	4885.15	0.90	0.57	2.09	0.17
					Specificity = 90%	13,743.13	0.42	0.90	4.27	0.64
	0—2 vs 3	12.3%	0.82 (0.77—0.87)	< 0.0001	Youden’s index	6874.85	0.91	0.65	2.57	0.14
					Sensitivity = 90%	6906.17	0.90	0.65	2.52	0.17
					Specificity = 90%	15,635.01	0.35	0.90	3.56	0.72

*KTRs* kidney transplant recipients, *CKDs* chronic kidney diseases, *AUC* area under the curve, *PLR*, positive likelihood ratio, *NLR* negative likelihood ratio

Similarly, in the native CKD cohort, sTIM-3 showed robust performance for identifying fibrosis stages 0 vs. 1–3, 0–1 vs. 2–3, and 0–2 vs. 3, with AUCs of 0.77 (0.73–0.82), 0.82 (0.77–0.86), and 0.82 (0.67–0.87), respectively (all *P* < 0.0001). Optimal cutoffs were identified at 6,766.99, 8,243.67, and 6,874.85 pg/mL. Performance characteristics at fixed 90% sensitivity or specificity for the CKD cohort are further detailed in Table 3. Collectively, these results indicate that plasma sTIM-3 effectively discriminates between progressive stages of renal fibrosis in both transplant and native kidney disease settings.

### Association of sTIM-3 with graft failure and eGFR decline in KTRs

**Fig. 4:**
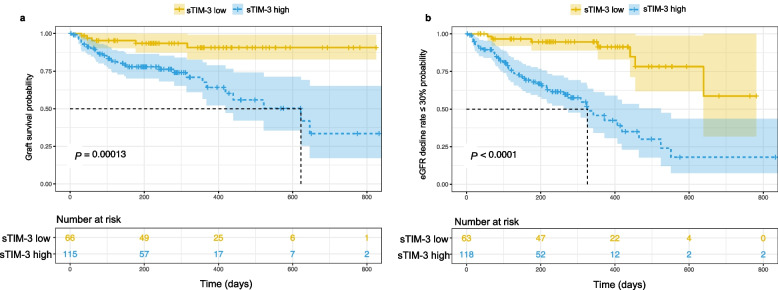
Kaplan–Meier survival curves for graft failure (**a**) and eGFR decline rate > 30% (**b**) in KTRs with indication biopsies according to the sTIM-3 levels. Note: In Fig. 4**a**, sTIM-3 low refers plasma sTIM-3 level < 5198.73 pg/mL; sTIM-3 high refers plasma sTIM-3 level ≥ 5198.73 pg/mL. In Fig. 4**b**, sTIM-3 low refers plasma sTIM-3 level < 5060.48 pg/mL; sTIM-3 high refers plasma sTIM-3 level ≥ 5060.48 pg/mL

To evaluate the prognostic value of sTIM‑3 for adverse outcomes in KTRs, we analyzed the association between pre‑biopsy sTIM‑3 levels and subsequent graft failure as well as rapid renal function decline using Kaplan–Meier curves, log-rank tests and Cox proportional hazards regression methods. Among KTRs who underwent allograft biopsy, 38 developed graft failure and 57 experienced rapid eGFR decline (defined as a > 30% reduction from pre-biopsy baseline) during follow-up. Stratified log-rank tests and Kaplan–Meier curves by optimal cut-off values revealed significant differences in graft failure (Fig. 4a) and eGFR decline (Fig. 4b) between sTIM-3 low and sTIM-3 high groups. Univariable (Table S2) and multivariable Cox regression confirmed that plasma sTIM-3 was significantly associated with both outcomes (Table 4). These associations remained significant after adjusting for recipient characteristics (age and sex) and histological features [IF/TA and interstitial inflammation (non-fibrotic cortex)]. Notably, upon further adjustment for baseline eGFR and urine protein, the association with graft failure was lost, whereas the predictive value for rapid eGFR decline persisted independently. These findings suggest that sTIM‑3 may be a significant predictor of adverse outcomes in KTRs, with its prognostic value for graft failure being partially dependent on renal function, while its association with functional decline remains independent of eGFR and urine protein.

**Table 4:** Cox regression analyses of the association of sTIM-3 with graft failure and eGFR decline in KTRs

	**Graft failure**		**eGFR decline rate > 30%**	
**Models**	**HR (95%CI)**	***P* value**	**HR (95%CI)**	***P* value**
sTIM-3 (binary)	5.28 (2.05—13.61)	0.001	6.63 (2.98—14.76)	< 0.0001
Model 1	5.29 (2.05—13.65)	0.001	6.77 (3.03—15.12)	< 0.0001
Model 2	5.20 (2.00—13.50)	0.001	6.63 (2.97—14.83)	< 0.0001
Model 3	3.97 (1.48—10.71)	0.006	5.08 (2.21—11.70)	0.0001
Model 4	2.26 (0.78—6.53)	0.134	3.02 (1.21—7.56)	0.018

Model 1 adjusted for age; Model 2 adjusted model 1 for sex; Model 3 adjusted model 2 for IF/TA and interstitial inflammation (non-fibrotic cortex); Model 4 adjusted model 3 for eGFR and urine protein

## Discussion

In this multi-cohort study, we systematically evaluated the clinical utility of plasma sTIM-3 as a diagnostic and prognostic biomarker across a broad spectrum of kidney diseases, integrating data from biopsy-confirmed KTRs and native CKD cohorts. Our findings yield several major insights. First, we validated that elevated sTIM-3 consistently correlates with impaired renal function across diverse etiologies, extending our previous observations in KTRs to native CKD populations (including IgAN, MN, DKD, HRD and ANCA-GN). Second, we demonstrated that sTIM‑3 is independently associated with the severity of IF/TA, a relationship significantly modulated by renal function as evidenced by the consistent sTIM‑3 × eGFR interaction. Third, we established that pre‑biopsy sTIM‑3 levels predict graft failure and rapid eGFR decline in KTRs, though its prognostic value for graft failure is attenuated after adjusting for eGFR. Fourth, we observed an intriguing paradoxical finding in MN patients, who exhibited higher sTIM‑3 levels despite preserved eGFR compared to IgAN patients, which we discuss in detail below. Collectively, these results position sTIM‑3 as a promising non‑invasive biomarker that reflects fibrotic kidney damage and adverse outcomes across both native and transplant‑related kidney diseases.

Membrane TIM-3 consists of IgV-like domain, a mucin domain, transmembrane domain and cytoplasmic tail. It is widely expressed on both innate and adaptive immune cells and has been shown to play critical immunoregulatory roles in various diseases [15]. Its soluble form, sTIM-3, comprises only the extracellular portion (IgV-like domain and mucin domain) and has demonstrated significant potential as a biomarker in multiple pathological conditions, including malignancies, infectious diseases, and autoimmune disorders [10]. The association between sTIM‑3 and renal function observed in our expanded cohorts aligns with and significantly extends prior work [13, 14, 16]. Our previous studies in KTRs demonstrated a negative correlation between sTIM‑3 and eGFR [13, 14], a finding now corroborated across multiple native CKD etiologies. This consistency suggests that the relationship is not merely a consequence of alloimmune activation but reflects a fundamental pathophysiological link between sTIM‑3 and kidney injury. Mechanistically, sTIM‑3 is generated via ectodomain shedding of membrane TIM‑3 by ADAM10/17 [9, 11], and its elevation in kidney disease may arise from increased shedding from activated immune cells or injured renal parenchymal cells. Notably, our longitudinal data revealed that sTIM‑3 declines more slowly than the rate of eGFR recovery post-transplantation. This suggests that sTIM‑3 may capture ongoing subclinical tissue remodeling that may persist even after functional improvement. This hypothesis warrants further investigation. A particularly intriguing finding is the paradoxical behavior of sTIM-3 in patients with native MN compared to those with IgAN. Although an inverse correlation between sTIM-3 and eGFR was observed within each subcohort, MN patients exhibited significantly higher sTIM-3 levels despite having better-preserved eGFR than IgAN patients. While MN and IgAN are both common primary glomerulopathies, their distinct pathophysiological mechanisms may underlie this discrepancy [17, 18]. The currently unexpected and inexplicable observation, whether related to differential immunological activation, variations in disease stage distribution, or histopathological heterogeneity between the two diseases, requires further clinical and experimental investigation.

The identification of non-invasive biomarkers capable of reflecting kidney histopathological changes is of paramount clinical importance. In this study, we comprehensively analyzed the associations between sTIM-3 and a wide range of histopathological features. A central finding is the robust and independent association between sTIM‑3 and IF/TA severity, which demonstrates significant diagnostic potential for moderate‑to‑severe kidney fibrosis across both KTR and native CKD cohorts. This association remained significant after adjusting for eGFR and other confounders, and the significant sTIM‑3 × eGFR interaction indicates that the relationship strengthens as renal function declines. These clinical observations are supported by experimental evidence. Data from the Human Protein Atlas and animal models demonstrate TIM-3 expression on renal resident cells, with particularly high expression on proximal tubule cells of both human and murine kidneys [7, 8]. Furthermore, TIM-3 is constitutively expressed on monocytes and macrophages, which are the key cellular mediators of renal inflammation and fibrosis [19]. A growing body of evidence has shown that TIM-3 is significantly upregulated in renal parenchymal cells and infiltrating monocytes/macrophages in both acute and chronic kidney injury models [6–8], consistent with clinical reports of elevated TIM-3 expression in kidney tissues from patients with IgAN and lupus nephritis [20, 21]. Collectively, these data suggest that kidney injury triggers TIM-3 overexpression in multiple cell types, which likely serves as the cellular source of elevated sTIM-3 levels observed in advanced fibrosis. This mechanistic link between proximal tubular injury, sustained TIM-3 expression, and progressive tissue remodeling provides a pathophysiological rationale for sTIM-3 as a potential surrogate marker of IF/TA.

Our previous work revealed that pre-transplant and post-1M sTIM-3 levels predicted infection [22], and composite adverse outcomes [23] within two years after kidney transplantation. These findings, together with the established role of IF/TA as a key driver of kidney disease progression, led us to hypothesize that sTIM-3 might be associated not only with functional decline but also with underlying fibrotic pathology and long-term adverse outcomes. In the current study, we evaluated the prognostic potential of pre-biopsy sTIM-3 for graft failure and rapid eGFR decline in KTRs. However, these analyses could not be extended to patients with native CKD due to an insufficient number of adverse events during the follow-up period, which would have compromised statistical reliability. Elevated sTIM‑3 levels at the time of biopsy significantly predicted both graft failure and rapid eGFR decline, with associations remaining robust after adjusting for recipient demographics and histological features. However, the attenuation of the graft failure association after adjustment for eGFR warrants careful interpretation. Two non-mutually exclusive explanations may account for this attenuation. First, the modest number of graft failure events may have limited statistical power, potentially masking a smaller but independent effect of sTIM-3. Second, this finding suggests that prognostic information provided by sTIM-3 for hard clinical endpoints is partially mediated by its correlation with renal function, and that sTIM‑3 may not offer substantial incremental prognostic value beyond eGFR for predicting graft loss. In contrast, its association with rapid eGFR decline remained independent of eGFR, indicating that sTIM‑3 may capture dynamic functional deterioration not fully explained by baseline function. Importantly, the primary strength of sTIM‑3 may lie not in its superiority over eGFR for prognosis, but in its ability to reflect underlying fibrotic pathology. Thus, sTIM‑3 should be viewed as a complementary biomarker that bridges the gap between functional assessment and structural histopathology, particularly for non‑invasive fibrosis evaluation.

From a clinical applicability perspective, sTIM‑3 measurement via ELISA is relatively inexpensive, readily accessible in clinical laboratories, and amenable to automation, positioning it as a potentially cost‑effective adjunct to existing biomarkers. Unlike eGFR and proteinuria, which primarily reflect glomerular function and injury, sTIM‑3 offers complementary information by specifically capturing tubulointerstitial fibrosis. Our longitudinal data suggest that peri‑transplant baseline measurements and annual or eGFR‑triggered monitoring in CKD patients may represent rational sampling strategies, although optimal timing and frequency require prospective validation. In addition, before clinical implementation, several prerequisites must be addressed: standardization of assays across platforms, establishment of reference ranges in diverse populations, and demonstration of incremental value in prospective management studies.

Despite these promising findings, several limitations should be noted in this study. First, we acknowledge that the modest number of graft failure events in our KTR cohort warrants caution. Although post‑hoc power calculations confirmed > 90% power to detect the observed hazard ratios (HR > 5.0) (data not shown), the limited event count precluded extensive subgroup analyses stratified by eGFR, and we cannot definitively determine whether the loss of independent prognostic value reflects true biological dependence or insufficient power. Larger multicenter studies with longer follow‑up and greater event numbers are essential to validate these findings and to establish whether sTIM‑3 provides independent prognostic information in well‑powered cohorts. Second, the single‑center design and exclusive enrollment of Chinese patients restrict the generalizability of our findings to other ethnic and geographic populations; genetic, environmental, and lifestyle factors may influence sTIM‑3 levels and their association with kidney disease progression. External validation in multi‑ethnic, international cohorts is essential before sTIM‑3 can be considered a broadly applicable biomarker for clinical use. Third, while we adjusted for numerous confounders in multivariable models, residual confounding cannot be excluded, particularly given the observational design. Fourth, the relatively short follow‑up in the native CKD cohort precluded the assessment of sTIM‑3's prognostic value for long‑term CKD progression, which remains a critical question for future research. Fifth, the mechanistic basis for the paradoxical relationship between sTIM-3 and eGFR in MN and IgAN patients remains to be fully elucidated, warranting further investigation into disease-specific biological pathways. Finally, although we included diverse CKD etiologies, some groups (e.g., HRD, ANCA-GN) had relatively small sample sizes, limiting the precision of subgroup estimates.

## Conclusion

In conclusion, this study provides comprehensive evidence that plasma sTIM-3 is a promising and robust biomarker that reflects the severity of renal functional impairment and chronic histopathological damage across a wide spectrum of kidney diseases. Its consistent associations with renal function, IF/TA severity, and clinical prognosis support its potential utility as a non-invasive marker for fibrosis assessment and risk stratification. Future work should focus on prospective validation in multi‑ethnic populations, elucidation of underlying mechanisms, and evaluation of whether sTIM‑3‑guided interventions can improve clinical outcomes in patients with progressive kidney disease.

## Materials and methods

### Study population

This study included three cohorts of participants recruited from West China Hospital of Sichuan University between June 2023 and December 2025, with follow-up continuing until January 2026. The KTR cohort comprised 256 adult KTRs who underwent transplantation at our center. Patients were eligible if plasma samples were available within 48 h prior to kidney biopsy (*n* = 219) or during routine follow-up for those with stable renal function (*n* = 37). Exclusion criteria included multi-organ transplantation, active infection at sample collection, malignancy within the past five years, or incomplete clinical data. The CKD cohort consisted of 442 adult patients diagnosed with CKD who underwent native kidney biopsy at our center, with plasma samples collected within 48 h prior to the procedure. Patients were excluded if they presented with acute kidney injury requiring emergency dialysis, had active infection or malignancy, or had incomplete clinical or histopathological data. The longitudinal KTR-dyna cohort enrolled 44 adult KTRs who completed one year of regular follow-up at our hospital. Patients who experienced graft loss within the first month post-transplantation were excluded. HCs included 80 adults who underwent health examinations at our center and had normal laboratory and imaging results. Individuals with any known history of chronic illness or current medication use were excluded. Clinical and laboratory data were extracted from the hospital information system and the laboratory information system, respectively. Laboratory data included complete blood count, kidney and liver function, lipid profile, and urine protein. We used creatinine-based eGFR to reflect kidney function according to Chronic Kidney Disease Epidemiology Collaboration equation (CKD-EPI). This study was approved by the institutional review board of West China Hospital, and written informed consent was obtained from each participant before enrollment. This study was conducted in accordance with the Declaration of Helsinki.

### Study design

To verify the association between sTIM-3 and kidney function, patients in both KTR and native CKD cohorts were grouped into three subgroups when eGFR was greater than 60, between 30 and 60 and less than 30 mL/min/1.73m^2^ for more than 3 months. In the KTR-dyna cohort, sTIM-3 concentrations were measured pre-transplantation and at designated post-transplantation follow-up visits.

To investigate the role of sTIM-3 in differentiating primary kidney disease etiologies and stages, we analyzed KTRs and patients with native CKD who underwent kidney biopsy. Allograft pathology of 219 KTRs was evaluated according to Banff 2022 classification [24]. KTRs were classified as having ABMR (*n* = 61), new onset or recurrence of IgAN (*n* = 63), mixed types (defined as allograft biopsies exhibiting overlapping features of two or more distinct diagnostic entities that cannot be assigned to a single category, *n* = 21), glomerular or tubular changes (not otherwise specified, NOS, *n* = 55) and others (including BK polyomavirus infection, T cell mediated rejection and recurrent glomerulonephropathy other than recurrent IgAN, *n* = 19). In the CKD cohort, kidney pathology was assessed using histomorphology, immunofluorescence and electron microscopy. Patients were classified as having MN (*n* = 122), IgAN (*n* = 165), DKD (*n* = 85), HRD (*n* = 13), ANCA-GN (*n* = 20) and others (*n* = 37). MN lesions were staged (I-IV) following Ehrenreich-Churg classification criteria [25], IgAN severity was graded (I-V) according to the Lee Grading system [26] and DKD severity was classified based on 2010 Tervaert classification [27].

To study the association between sTIM-3 and histopathological lesion indicators, histopathological feature data were collected through a review of clinical histology reports. This included data on IF/TA, interstitial inflammation (non-fibrotic cortex), tubulitis, global glomerulosclerosis, mesangial matrix expansion, glomerulitis, arteriolar sclerosis, arteriolar hyalinosis, peritubular capillaritis and C4d deposition. Qualitative and quantitative histopathological features were converted into ordinal categories for further comparisons where possible.

To explore the prognostic value of sTIM-3 in predicting adverse outcomes in kidney diseases, sTIM-3 was quantified at pre-biopsy baseline to assess subsequent graft failure (defined as eGFR < 15 mL/min/1.73m^2^, dialysis, or re-transplantation) and rapid eGFR decline (defined as an eGFR decline rate > 30%).

### Sample collection and sTIM-3 concentration determination

Plasma samples were collected within 48 h prior to kidney biopsy, and aliquots were stored at −80℃ in both KTR and CKD cohorts. For the KTR-dyna cohort, plasma samples were collected longitudinally at the following time points: pre-transplantation (pre-KT), postoperative day 7 (Post-7D), month 1 (Post-1 M), 2 (Post-2 M), 3 (Post-3 M), 6 (Post-6 M) and 12 (Post-12 M). sTIM-3 concentrations were quantified using the commercially available ELISA kit (Cat. No. JL19175; Jianglai Biotechnology, Shanghai, China) according to the manufacturer’s instructions.

### Statistical analyses

The data were presented as mean ± SD, median (interquartile range), or number (percentage) as appropriate. Student’s t-test and Mann–Whitney U test were applied to compare continuous variables with normal and skewed distributions, respectively. Chi-square or Fisher’s exact tests were utilized to compare categorical variables between groups. Spearman correlation coefficients were used to determine associations between markers. Univariable and multivariable generalized linear regression analyses were used to evaluate the associations of sTIM-3 level with IF/TA severity, adjusted for demographic data, renal function and other laboratory markers to identify other independent potential markers of IF/TA in both CKD and KTR cohorts. ROC curve was constructed to assess the diagnostic performances of sTIM-3 for IF/TA severity. Diagnostic statistics and sTIM-3 cut-off values for increasing pairwise fibrosis stages (0 vs. 1—3, 0—1 vs. 2—3 and 0—2 vs. 3) were estimated with cutoff criteria set at Youden index (sensitivity + specificity −1), sensitivity fixed at approximately 90%, and specificity fixed at approximately 90%. Positive likelihood ratio (PLR) and negative likelihood ratio (NLR) were calculated as follows: PLR = sensitivity/(1-specificity), NLR = (1-sensitivity)/specificity. Kaplan–Meier curves and log-rank tests were conducted to visualize and assess the significance of differences in graft survival and eGFR preservation between high and low sTIM-3 levels in the KTR cohort. Cox proportional hazards regression analyses were performed to determine the independent association of sTIM-3 with allograft outcomes. Variables were selected for multivariable Cox regression based on clinical relevance established in our previous literature (e.g., recipient age, gender and urine protein) [28] and statistical significance in univariable analyses (*P* < 0.10). All statistical analyses were completed with SPSS software (version 23.0, SPSS Inc., Chicago IL, USA) and R software (version 4.2.3 https://cran.r-project.org). R packages “Hmisc”, “corrplot”, “PerformanceAnalytics” were used in this study to generate correlation matrix. A two-sided *P* value < 0.05 was considered statistically significant.

## Supplementary Information

Supplementary Material 1.

## Data Availability

The data supporting the findings from this study are available within the article and its supplementary materials. Further inquiries can be directed to the corresponding author.
