# Supplementary material for: Unlocking the roles of plasma soluble T-cell immunoglobulin and mucin domain-containing protein 3 in kidney diseases: findings from native and allograft biopsy cohorts
Source: Mol Biomed. 2026 May 27;7:77. doi: 10.1186/s43556-026-00469-6 (PMC13216427; doi:10.1186/s43556-026-00469-6)
Supplement: Supplementary file 1 — Supplementary Material 1. [file 43556_2026_469_MOESM1_ESM.docx]

**Unlocking the roles of plasma soluble T-cell immunoglobulin and mucin domain-containing protein 3 in kidney diseases: findings from native and allograft biopsy cohorts**

Yamei Li^1,2#^, Hua Zhang^3,4#^, Yangjuan Bai^1,2^, Huan Xu^1,2^, Yan Luo^3^, Dan Ye^3,5^, Xueqiao Wang^1,2^, Xingxin Gong^1,2^, Qu Yang^1,2^, Hanjing Liu^1,2^, Binqi Yang^6^, Zheyuan Zhang^1,2^, Yuxin Ye^1,2^, Yunfei An^1,2^, Xinhua Dai^1,2^, Lanlan Wang^1,2^*, Yunying Shi^6^*

1 Department of Laboratory Medicine/Clinical Laboratory Medicine Research Center, West China Hospital, Sichuan University, Chengdu, Sichuan province, China

2 Sichuan Clinical Research Center for Laboratory Medicine, Chengdu, Sichuan province, China

3 Department of Pathology, General Hospital of Western Theater Command, Chengdu, Sichuan province, China

4 Tissue Stress Injury and Functional Repair Key Laboratory of Sichuan Province, General Hospital of Western Theater Command, Chengdu, Sichuan province, China

5 College of Medicine, Southwest Jiaotong University, Chengdu, China

6 Department of Nephrology, West China Hospital, Sichuan University, Chengdu, Sichuan province, China

^#^Yamei Li and Hua Zhang contribute equally to this work

*** Correspondence to:** Yunying Shi, [yyshi0130@163.com](mailto:yyshi0130@163.com)**,** No.37 Guoxue Xiang, Wuhou district, Chengdu 610041, China.

Lanlan Wang, [wanglanlanhx@163.com](mailto:wanglanlanhx@163.com), No.37 Guoxue Xiang, Wuhou district, Chengdu 610041, China.

**Supplementary materials**

**Supplementary Table 1 The Baseline characteristics of KTR and CKD subcohorts**

|  | **KTRs** | | **Native CKDs** | | |
| --- | --- | --- | --- | --- | --- |
| **Characteristic** | **ABMR** | **New onset or recurrence of IgAN** | **MN** | **IgAN** | **DKD** |
| Number | 61 | 63 | 122 | 165 | 85 |
| Age (year) | 38 (30-51) | 37 (32-43) | 53 (42-59) | 35 (29-44) | 35 (29-44) |
| Male, n (%) | 47 (77.05%) | 49 (77.78%) | 68 (55.74%) | 70 (42.42%) | 63 (74.12%) |
| Living donor, n (%) | 46 (75.41%) | 54 (85.71%) | NA | NA | NA |
| HLA-mismatch (A, B, DR, DQ) | 4 (4-7) | 4 (3-4) | NA | NA | NA |
| Post-transplant time (years) | 3.14 (1.13-5.94) | 4.39 (2.19-8.21) | NA | NA | NA |
| Trough concentration of tacrolimus (ng/mL) | 5.61 (4.64-7.17) | 5.51 (4.88-6.51) | NA | NA | NA |
| SCR (μmol/L) | 169.00 (140.00-258.00) | 149.00 (116.00-208.50) | 72.50 (61.00-88.00) | 94.00 (70.00-123.00) | 100.00 (150.00-209.00) |
| eGFR (mL/min/1.73m2) | 38.89 (22.21-53.17) | 45.88 (34.51-61.41) | 94.79 (78.23-106.96) | 78.91 (56.69-100.82) | 43.99 (23.93-69.07) |
| Serum urea, mmol/L | 11.30 (7.90-18.90) | 9.30 (6.65-11.55) | 5.15 (4.10-6.50) | 5.40 (4.50-7.30) | 10.30 (7.10-13.10) |
| Serum cystatin C, mg/L | 2.16 (1.62-3.29) | 1.74 (1.42-2.08) | 1.04 (0.94-1.27) | 1.11 (0.93-1.34) | 1.64 (1.27-2.02) |
| Urine protein, n |  |  |  |  |  |
| 0 | 11 | 6 | 0 | 10 | 0 |
| +/- | 6 | 2 | 1 | 12 | 1 |
| + | 13 | 13 | 12 | 38 | 6 |
| ++ | 19 | 33 | 34 | 64 | 36 |
| +++ | 11 | 8 | 32 | 30 | 26 |
| ++++ | 1 | 1 | 43 | 11 | 16 |
| Urine protein-to-creatinine* ratio | 0.21 (0.06-0.36) | 0.15 (0.08-0.31) | 0.34 (0.16-0.77) | 0.11 (0.06-0.17) | 0.42 (0.28-0.78) |
| Ehrenreich-Churg classification |  |  |  |  |  |
| StageⅠ | NA | NA | 38 | NA | NA |
| StageⅡ | NA | NA | 76 | NA | NA |
| Stage Ⅲ | NA | NA | 8 | NA | NA |
| Lee Grading |  |  |  |  |  |
| Grade Ⅰ | NA | NA | NA | 1 | NA |
| Grade Ⅱ | NA | NA | NA | 16 | NA |
| Grade Ⅲ | NA | NA | NA | 105 | NA |
| Grade Ⅳ | NA | NA | NA | 20 | NA |
| Grade Ⅴ | NA | NA | NA | 3 | NA |
| Unknown | NA | NA | NA | 19 | NA |
| 2010 Tervaert classification |  |  |  |  |  |
| Class Ⅰ | NA | NA | NA | NA | 4 |
| Class Ⅱ | NA | NA | NA | NA | 13 |
| Class Ⅲ | NA | NA | NA | NA | 41 |
| Class Ⅳ | NA | NA | NA | NA | 16 |
| Unclassified | NA | NA | NA | NA | 11 |
| ALT, IU/L | 14.00 (9.00-18.00) | 15.00 (10.00-21.50) | 17.50 (13.00-25.00) | 14.00 (10.00-22.00) | 17.00 (11.50-24.00) |
| AST, IU/L | 15.00 (10.00-18.00) | 15.00 (12.50-20.00) | 19.00 (16.00-26.00) | 18.00 (15.00-22.00) | 19.00 (15.00-25.00) |
| Total cholesterol, mmol/L | 5.14 (4.27-5.87) | 5.33 (4.68-6.23) | 6.04 (4.78-7.24) | 4.60 (4.18-5.41) | 4.83 (3.71-6.02) |
| Triglyceride, mmol/L | 1.84 (1.15-2.28) | 1.87 (1.44-2.47) | 2.18 (1.50-3.08) | 1.53 (1.10-2.13) | 1.76 (1.25-2.67) |
| Hemoglobin, g/L | 118.85± 25.05 | 131.24 ± 23.69 | 130.18 ± 19.03 | 131.64 ± 18.75 | 116.56 ± 23.56 |
| Neutrophil-to-lymphocyte ratio | 3.83 (2.47-7.94) | 3.24 (2.33-6.06) | 2.30 (1.60-3.70) | 2.35 (1.76-2.89) | 2.79 (2.08-3.47) |

Note: *HLA*, human Leukocyte Antigen; *SCR*, serum creatinine; *eGFR*, estimated glomerular filtration rate; *UPCR*, Urine protein-to-creatinine; *ALT*, Alanine aminotransferase; *AST*, Aspartate aminotransferase; *NA*, not applicable. * In the ABMR subcohort, UPCR was missing in 67.2% of patients; In the new onset or recurrence of IgAN subcohort, UPCR was missing in 49.2% of patients.

**Supplementary Table 2 Univariable Cox hazardous regression analyses to select the potential factors associate with graft failure and eGFR decline in KTRs**

|  | **Graft failure** | | **eGFR decline rate >30%** | |
| --- | --- | --- | --- | --- |
| **Variables** | **HR (95%CI)** | **P value** | **HR (95%CI)** | **P value** |
| Age (years) | 1.00 (0.97-1.03) | 0.986 | 0.99 (0.97-1.02) | 0.994 |
| Male | 1.43 (0.63-3.26) | 0.392 | 1.49 (0.77-2.88) | 0.236 |
| Post-transplant time (years) | 0.93 (0.83-1.05) | 0.236 | 0.98 (0.90-1.06) | 0.583 |
| Deceased donor | 1.33 (0.65-2.76) | 1.334 | 1.48 (0.82-2.66) | 0.196 |
| sTIM-3 level (pg/mL) |  |  |  |  |
| sTIM-3 low* | 1 |  | 1 |  |
| sTIM-3 high* | 5.28 (2.05-13.61) | 0.001 | 6.63 (2.98-14.76) | <0.0001 |
| eGFR (mL/min/1.73m^2^) | 0.92 (0.89-0.94) | <0.0001 | 0.95 (0.93-0.97) | <0.0001 |
| Urine protein (n) |  |  |  |  |
| 0 | 1 |  | 1 |  |
| +/- | 0.78 (0.20-3.15) | 0.730 | 0.53 (0.14-1.97) | 0.346 |
| + | 0.94 (0.33-2.71) | 0.905 | 0.77 (0.32-1.87) | 0.563 |
| ++ | 0.66 (0.24-1.81) | 0.414 | 0.75 (0.34-1.68) | 0.483 |
| +++ | 1.65 (0.59-4.64) | 0.345 | 1.41 (0.60-3.31) | 0.433 |
| ++++ | 1.86 (0.38-9.30) | 0.448 | 1.83 (0.49-6.79) | 0.365 |
| Trough concentration of tacrolimus (ng/mL) | 0.95 (0.78-1.15) | 0.947 | 0.96 (0.82-1.13) | 0.604 |
| HLA mismatch | 1.11 (0.89-1.38) | 0.363 | 1.05 (0.86-1.28) | 0.623 |
| DSA at biopsy | 0.86 (0.39-1.91) | 0.709 | 0.76 (0.39-1.50) | 0.761 |
| C4d positive | 1.51 (0.76-2.99) | 0.241 | 1.06 (0.61-1.83) | 0.832 |
| Allograft biopsy results |  |  |  |  |
| Others, n (%) | 1 |  | 1 |  |
| Biopsy-proved rejection, n (%) | 0.97 (0.46-2.05) | 0.938 | 1.10 (0.57-2.12) | 0.772 |
| Recurrent glomerulonephropathy, n (%) | 0.55 (0.24-1.28) | 0.166 | 0.77 (0.39-1.52) | 0.448 |
| IF/TA | 1.03 (1.01-1.05) | 0.004 | 1.04 (1.02-1.06) | <0.0001 |
| Interstitial inflammation (non-fibrotic cortex) |  |  |  |  |
| i=0 | 1 |  | 1 |  |
| i=1 | 3.77 (1.41-10.11) | 0.008 | 2.86 (1.38-5.92) | 0.005 |
| i=2 | 4.37 (1.39-13.79) | 0.012 | 3.18 (1.29-7.84) | 0.012 |
| i=3 | 6.77 (1.95-23.50) | 0.003 | 8.27 (3.23-21.21) | <0.0001 |

Note: *HR*, hazard ratio; *95%CI,* 95% confidential interval; *eGFR*, estimated glomerular filtration rate; *HLA*, human leukocyte antigen; *DSA*, donor specific antibody; *IF/TA*, tubulointerstitial fibrosis and tubular atrophy.

**Supplementary Figure 1 Correlation matrices of sTIM-3 levels with renal function biomarkers in KTR and native CKD subcohorts**

a. ABMR subgroup (n=61); b. New onset or recurrence of IgAN subgroup (n=63); c. Native IgAN subcohort (n=165); d. Native MN subcohort (n=122); e. DKD subcohort (n=85). Note: SCR, serum creatinine; Upro, urine protein; UPCR, urine protein-to-creatinine ratio; BUN, blood urea nitrogen; CYC, Cystatin C; UA, uric acid

**Supplementary Figure 2 Comparative analyses of sTIM-3 and eGFR levels across native IgAN, MN and DKD disease stages**

a, d. sTIM-3 (a) and eGFR (d) levels in native IgAN patients stratified by Lee Grading system;

b, e. sTIM-3 (b) and eGFR (e) levels in native MN patients stratified by Ehrenreich-Churg classification criteria;

c, f. sTIM-3 (c) and eGFR (f) levels in native DKD patients stratified by 2010 Tervaert classification.

**Supplementary Figure 3 Association between plasma sTIM-3 and kidney histological lesions in MN patients.**

The scatter plots depict variations of sTIM-3 levels across different severity grades of kidney histological lesions in native IgAN, MN and DKD patients, including IF/TA (a, g, m), interstitial inflammation (non-fibrotic cortex) (b, h, n), arteriolar hyalinosis (c, i, o), global glomerulosclerosis (d, j, p) mesangial matrix expansion (e, k, q) and glomerulitis (f, l, r).

**Supplementary Figure 4 Correlation analyses between sTIM-3, eGFR and IF/TA areas**

Scatter plots with Spearman’s rank correlation coefficients showing the positive correlations between sTIM-3 and IF/TA areas in KTRs (a), ABMR subcohort (b), new onset or recurrence of IgAN subcohort (c), native CKD cohort (g), IgAN subcohort (h) and MN subcohort (i) and DKD subcohort (j). Scatter plots with Spearman’s rank correlation coefficients showing the negative correlations between eGFR levels and IF/TA areas in KTRs (d), ABMR subcohort (e), new onset or recurrence of IgAN subcohort (f), native CKD cohort (k), IgAN subcohort (l) and MN subcohort (m) and DKD subcohort (n).
